# Mortality Rate and Years of Life Lost Due to Burns in Southern Iran During 2004–2019: A Population-Based Study

**DOI:** 10.34172/aim.2023.31

**Published:** 2023-04-01

**Authors:** Alireza Mirahmadizadeh, Leila Moftakhar, Seyed Sina Dehghani, Jafar Hassanzadeh, Seyed Parsa Dehghani, Habibollah Azarbakhsh

**Affiliations:** ^1^Non-Communicable Diseases Research Center, Shiraz University of Medical Sciences, Shiraz, Iran; ^2^Student Research Committee, Shiraz University of Medical Sciences, Shiraz, Iran; ^3^School of Medicine, Shiraz University of Medical Sciences, Shiraz, Iran; ^4^Research Center for Health Sciences, Institute of Health, Department of Epidemiology, Shiraz University of Medical Sciences, Shiraz, Iran

**Keywords:** Burns, Mortality, Trend, Years of life lost, Joinpoint regression

## Abstract

**Background:**

Burns constitute one of the most important etiologies of infection and mortality worldwide, with the most significant number of cases in low- and middle-income countries. This is a cross-sectional study on deaths due to burns in southern Iran.

**Methods:**

In this study, data on all deaths due to burns in southern Iran between 2004 and 2019 was extracted from the population-based Electronic Death Registry System (EDRS). The Joinpoint Regression method was used to examine the trend of crude mortality rate, standardized mortality rate, and years of life lost (YLL) rate. In order to measure YLL, the number of deaths and life expectancy for different age and gender groups were used, for which the standard life table was considered.

**Results:**

During this study, 2175 deaths due to burns occurred, 50.6% (1106 cases) of which were in men and 38.7% (841 cases) were in the 15-29 age group. The crude and the standardized mortality rate had a decreasing trend during the study years. The total number of YLL was 25260 (0.8 per 1000) in men, 25,785 (0.8 per 1000) in women, and 51,045 (0.8 per 1000) in both genders during the 16 years of the study.

**Conclusion:**

Considering the high mortality rate in the 15-29 age group, which consists of the active and productive labor force, necessary actions are needed in order to improve safety equipment and to make the workplace safe.

## Introduction

 Burns constitute one of the most important etiologies of infection and mortality worldwide, with the most significant number of cases in low- and middle-income countries.^1^ More than 300 000 deaths occur annually worldwide due to burns.^2^ Burning and its adverse effects on organ health can lead to open wounds, death, disability, severe emotional complications, and a high economic burden.^3^ Burns are annually responsible for 1% of the world’s burden of diseases, more than 7 million damages, and 18 million disability adjusted life years (DALYs) all over the world.^4^ Burns impose a high cost on the health system; for example, burning has a cost of 3000 to 5000 dollars for each patient in the United States.^5^ The annual incidence of burns is 110 per 100 000 in the Eastern Mediterranean region, with over 500 million people living in 22 countries. At the same time, the United States has the lowest annual incidence rate of 19 cases per 100 000, and southeastern Asian countries have the highest annual incidence rate of 243 per 100 000.^6^ The mortality rate of burn is high in Iran, a middle-high income country.^7^ The injuries due to burning cause a significant burden on the health system in Iran, such that it is the 8th cause of years of life lost (YLL) and the 13th cause of mortality and DALYs.^8^ Burns are one of the major concerns for the children’s health in Iran and the 2nd cause of death in this age group after car accidents.^9^

 The YLL is an importantcriterion for ranking the status of society health and evaluating its challenges. According to the report of the WHO, the value of 1 year of life is three times higher than the gross domestic product of each country.^10^ Because there has been no study to measure YLL due to burns in the Fars province, this study was designed to provide this missing information.

## Materials and Methods

 The present study is a cross-sectional study conducted in the Fars province. Between 2004–2019, we extracted all deaths due to burns based on gender, age, and the year of death according to the ICD-10 from the EDRS (Electronic Death Registry System) that is available in the Health Statistics Unit of Shiraz University of Medical Sciences. All deaths from different causes are reported to the Committee of Death Registration, and using EDRS, all local offices can choose a common way to report death, which will help to collect and interpret data at the international and national level. Information on new deaths can be collected as the users enter the data in the software and data analysis can be performed easily.^11^ This registry collects data from different sources including forensic medicine department, hospitals, and local health centers. As a result, the potential risks of error are reduced when using EDRS to analyze death data at a national or international level.

 The codes used in this study were X00-X19. In the population-based EDRS, we used all the resources for diagnosing, registering and collecting information about death, then the duplicated cases and the cases of self-burn were excluded from the study. Regarding the deaths occurring outside health centers and hospitals, especially in remote rural areas, questionnaires of verbal autopsy are used in order to improve the quality of information about the causes of deaths, administered by doctors.^12^ Because different sources report deaths to the committee of death registration, the possibility of error is reduced.

 In this study, sampling was not performed, because the purpose was to calculate the YLL due to burns. We used a population-based electronic death registration system. In Iran, trained doctors first report deaths and then the causes of death are coded according to the International Diseases Classification (ICD) and the national protocol. Afterwards, cemeteries, hospitals, local health centers, and forensic organizations report these data monthly to the Death Registration Committee.^13^ As a result, the probability of underestimation or overestimation of death cases is reduced.

 In different places, data is checked with other sources. For example, the number of deaths is checked with Vital Horoscope in rural areas; also, other characteristics and number of deaths are checked with the civil registration bureau. Data from health centers and health houses in rural and urban areas, forensic medicine, hospitals, and cemeteries are transmitted to district health center, where the data is checked with civil registration. Data is sent 3-6 months to the provincial health center every and then annually to the Ministry of Health.^14^

 The total population of the Fars province was estimated based on the data from health centers and census done between 1996 and 2016 with the calculation of annual population growth rate. The standard population in 2013 for low- and middle-income countries was used for standardization.

###  Statistical Analysis

 First, the crude and standardized mortality rate of burns was calculated based on the gender and the year of death.

 Then, to calculate YLL, we used the table of standard age and determined life expectancy for different age and gender groups, in addition to the number of deaths due to burns; calculations were made for each gender and age group based on the following formula.^15^


$$YLL=N\;Ce^{\left(ra\right)}/{\left(\beta +r\right)}^{2}\;\left[e^{-\left(\beta +r\right)\left(L+a\right)}\;\left[-\left(\beta +r\right)\left(L+a\right)-1\right]\;-e^{-\left(\beta +r\right)a}\;\left[-\left(\beta +r\right)\;a-1\right]\right]$$

N = number of deaths in a specified age and gender group

 L = life expectancy of death cases again in that age and gender group

 r = Discounting Rate, which equals 0.03.

 β = a conventional rate in calculating age value which equals 0.04.

 C = an adjusted, constant value that equals 0.1658.

 a = the age at which death occurred

 e = a constant value considered as 2.71.

 Initially, YLL due to premature death related to burning was calculated for 18 age groups (0-4, 5-9–10-15, … and so on up to 85) and was then charted based on the data of the following age groups: 0-4, 5-14, 15-29, 30-44, 45-59, 60-70, 70-79 and + 80.

 The YLL analysis due to premature death related to burning was executed by the 2015 YLL template from the WHO in the EXCEL 2016 software.

 To check the trend of crude and standardized mortality and YLL rates for various years, Joinpoint regression was used on the basis of log-linear model. The motivations for the use of a log-linear model are limited data points and ease of interpretation. Unlike linear regression which is based on slope, the log linear regression is based on the APC (i.e. the rates change at a constant percent per year). It can also be used to compare trends across scales.^16^

 The generalized log-linear joinpoint regression model for the observations is: (x1, y1)… (XN, yN), where x1 < … < , X_N_ represent the variable of time, e.g. calendar year, and yi, i = 1, 2… N represents the annual rates as follows: log (yi) = E[yi|xi] + εi, where εi is the residual for the ith time, and the regression mean E[yi|xi] is defined as a succession of (n + 1) linear segments over the time interval [a,b].^17^

 In our analysis, X_i_ represented years between 2004 and 2019 and y_i_ is the annual burn YLL rate during this time.

 The final model is a set of joined log-linear segments between successive joinpoints, with each segment described by its short-term annual percentage change (APC).^18^

 Joinpoint regression analysis describes the trends of changing over successive segments of time and the decrease or increase within each part. The annual percent change (APC) describes the resulting line segment between joinpoints based on the average annual percent change (AAPC) and the slope of the line segment. We used constant variance (homoscedasticity) and uncorrelated in our analysis. Analysis of the trend was carried out using the Joinpoint Regression Program 4.9.0.0.

 The protocol of this study was reviewed and approved by the Ethics Committee of Shiraz University of Medical Sciences (SUMS). All aspects of this study complied with the SUMS moral code.

## Results

 During the 16 years of study (2004-2019), 2175 cases of death occurred due to burns in the Fars province. Of these, 50.6% (1101 cases) occurred in men and 38.7% (841 cases) occurred in the 15-29 age group.

 As demonstrated in Table 1, the crude mortality rate due to burns in men decreased from 4.8 per 100 000 in 2004 to 2.9 per 100 000 in 2019 (*P* for trend = 0.001); in women, it decreased from 5.6 per 100 000 in 2004 to 2.8 per 100,000 in 2019 (*P* for trend < 0.001). The standardized mortality rate in men decreased from 5.1 per 100,000 in 2004 to 2.8 per 100,000 in 2019 (*P* for trend < 0.001), while in women, it decreased from 5.0 per 100,000 in 2004 to 1.8 per 100,000 in 2019 (*P* for trend < 0.001) (Table 1).

**Table 1:** Crude and the Standardized Mortality Rate (per 100 000) and YLL due to Burns based on Gender and Age in the Fars Province

**Year**	**Number of Deaths**		**Crude Mortality Rate**		**ASR**^a^ ** (95% CI)**		**YLL**			
							**No.**		**(Per 1000)**	
	**Male**	**Female**	**Male**	**Female**	**Male**	**Female**	**Male**	**Female**	**Male**	**Female**
2004	90	99	4.8	5.6	5.1 (4.1-6.1)	5.0 (4.0-6.0)	2188	2503	1.2	1.4
2005	64	73	3.5	4.1	3.5 (2.7-4.3)	3.8 (2.8-4.8)	1464	1759	0.8	1.0
2006	91	95	4.9	5.3	4.7 (3.7-5.7)	5.4 (4.4-6.4)	2146	2230	1.2	1.2
2007	93	103	5.0	5.6	4.8 (3.8-5.8)	4.9 (3.8-5.9)	2038	2584	1.1	1.4
2008	98	132	5.2	7.1	4.8 (3.8-5.8)	6.1 (4.9-7.3)	2370	3328	1.3	1.8
2009	81	77	4.3	4.1	4.0 (3.1-4.9)	3.6 (2.6-4.6)	1759	1734	0.9	0.9
2010	86	81	4.5	4.3	4.2 (3.3-5.1)	3.9 (2.9-4.9)	2049	1961	1.1	1.0
2011	56	55	2.9	2.9	2.5 (1.8-3.3)	2.6 (1.9-3.4)	1320	1317	0.7	0.7
2012	91	63	4.6	3.2	4.4 (3.4-5.4)	3.1 (2.3-3.9)	2010	1372	1.0	0.7
2013	48	57	2.4	2.9	2.0 (1.4-2.6)	2.6 (1.8-3.4)	1145	1431	0.6	0.7
2014	45	49	2.2	2.5	2.2 (1.5-2.9)	2.4 (1.7-3.1)	1012	1151	0.5	0.6
2015	53	53	2.6	2.6	2.3 (1.6-3.0)	2.2 (1.5-2.9)	1217	1235	0.6	0.6
2016	46	32	2.2	1.6	2.1 (1.4-2.8)	1.6 (1.0-2.2)	1063	690	0.5	0.3
2017	41	39	2.0	1.9	2.0 (1.4-2.6)	2.1 (1.5-2.7)	960	925	0.5	0.5
2018	58	26	2.8	1.3	2.3 (1.6-3.0)	1.3 (0.8-1.8)	1243	586	0.6	0.3
2019	60	40	2.9	2.0	2.8 (2.0-3.6)	1.8 (1.2-2.4)	1276	979	0.6	0.5
Total	1101	1074	3.5	3.5	3.3 (3.1-3.5)	3.2 (3.0-3.4)	25260	25785	0.8	0.8
*P* Value for trend^b^	—	—	0.001	< 0.001	< 0.001	< 0.001	—	—	< 0.001	< 0.001

^a^Direct standardization method and using the 2013 Segi standard populations of low- and middle-income countries. ^b^Null hypotheses (H0): trend is constant, alternative hypotheses (H1): trend isn’t is not constant.

 The total YLL due to burns during the 16 years of study was 25,260 (0.8 per 1000) in men, 25,785 (0.8 per 1000) in women and 51,045 (0.8 per 1000) in both genders (gender ratio: women / men = 1.02) (Table 1).

**Figure 1:**
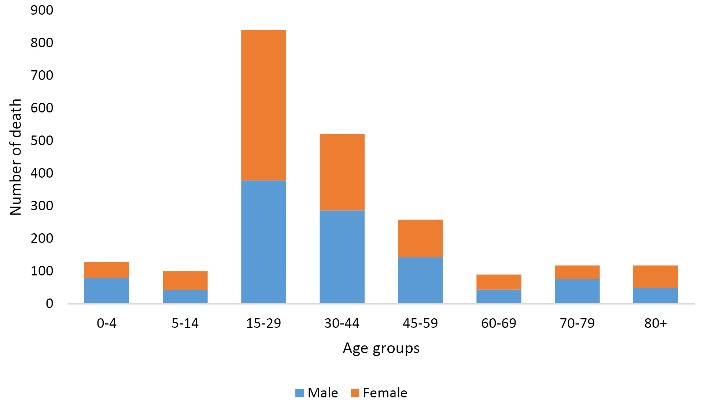
Number of Deaths due to Burns Based on the Age Groups and Gender.

 The greatest number of deaths in both genders pertained to the 15-29 age group; the lowest number of deaths pertained to the 5-14 age group in men, and the 70-79 age group in women (Figure 1).

**Figure 2:**
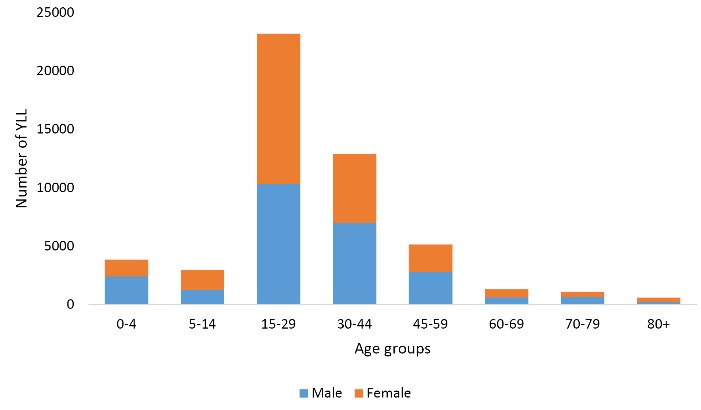
YLL Due to Burns Based on the Age Groups and Gender.

 The greatest number of YLL in both genders was found in the 15-29 age group and the lowest number of YLL in both genders was observed in 80 + age group (Figure 2).

**Figure 3:**
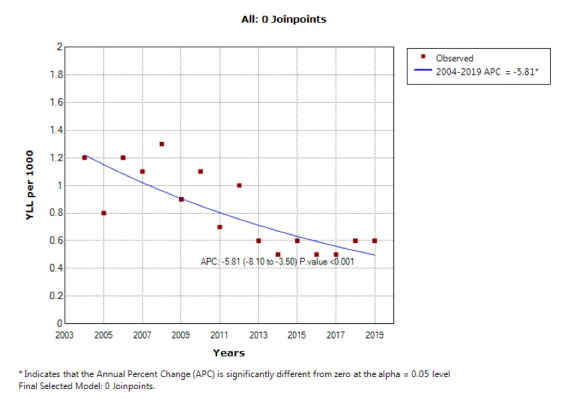
Trend of Years of Life Lost Due to Burns in Men During 2004–2019.

**Figure 4:**
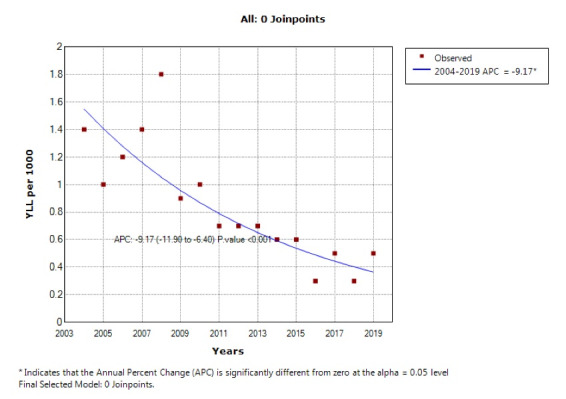
Trend of Years of Life Lost Due to Burns in Women During 2004–2019.

 According to the joinpoint regression analysis, the 16-year trend of YLL rate was decreasing due to premature mortality: the APC was -5.8% (95% CI -8.1 to -3.5, *P* < 0.001) for males, -9.2% (95% CI -11.9 to -6.4, *P* < 0.001) for females, and -7.8% (95% CI -10.0 to-5.6, *P* < 0.001) for both sexes. The model did not show any joinpoint; hence, the AAPC was the same as APC (Figures 3 and 4).

## Discussion

 The current study was done in order to determine the mortality rate and the YLL due to burning during 16 years in southern Iran. Burns are one of the leading social hygiene threats in developing countries and one of the most destructive social health crises worldwide.

 During the 16 years of our study, 2175 deaths due to burns occurred, 50.6% (1106 cases) of which were in men, and 38.7% (841 cases) were in the 15-29 age group. Although the mortality rate due to burning was approximately equal in men and women in our study, some studies reported a higher rate in men.^19-24^ In comparison, some reported a higher rate in women.^7^ Saeman and colleagues also reported that 2/3 of burn deaths occur in men.^25^ In a study done in Tokyo, there was no significant relationship between gender and the mortality rate of burns.^26^ Despite this, we should consider that men work in more threatening workplaces and are often in contact with flammable objects. At the same time, there is the possibility of insufficiency of the protective and safety measures. Moreover, men use flammable objects more often in their leisure time.^27,28^ On the other hand, women deal with fire and hot liquids more often while cooking, which puts them at risk of burns if enough care is not taken. Furthermore, there might be lack of appropriate cooking equipment for women in countries with low financial and social status. Besides that, a special form of women’s costumes (long dresses and covered appearance), which is usually a cultural issue, could lead to burns with minimal contact.^7^ Nevertheless, gender differences in different societies indicate the performance and physical activities and risk-taking in the workplace and leisure and the cultural issues of each country.^24^

 When we considered the age in our study, the highest mortality rate in women and men was seen in the 15-29 age group, which consists of the active and productive labor force, as reported by Sadeghian and colleagues.^7^ Panjeshahin et al reported that the highest mortality rate due to burning was in the 20-29 age group in southwestern Iran.^21^ However, the highest mortality rate was seen in different age groups in other studies, as some studies stated that children and elders are at the highest risk of burns.^21-24^ According to Kumar et al, 89% of the deaths occurred in the 10-49 age group.^29^ As discussed previously, there are different reports on the age group with the highest mortality rate, which might be due to numerous reasons such as the demographic structure of societies, demographic characteristics, and socioeconomic characteristics.^7^ Nevertheless, it should be considered that children and elders have a worse prognosis and higher mortality rate. In children, this is due to their weaker immune system and more contact with soup, milk and other hot liquids, while in elders, it is due to less mobility, less self-care ability, and underlying diseases.^28^ Nevertheless, generally, the 15-29 age group was identified as a high-risk group in our study, which could be the result of occupational contact and non-compliance with safety regulations in the workplace which highlights the need for training in order to prevent burns and dangerous encounters.

 In our study, the trend of crude and the age-standardized mortality rate decreased in both sexes during the study period. Multiple studies reported a similar trend.^19,20,22,27,30^ In another study, the standardized mortality rate decreased from 5.97 per 100 000 in 1990 to 1.74 per 100 000 in 2015.^7^ The mortality rate decreased from 7.03 to 0.53 in 5 years in Chile,^31^ and it decreased from 1.4 to 0.97 per 100 000 in Colombia between 2000-2009.^32^ A decreasing trend was also observed in the Netherlands for 17 years.^33^ On the other hand, Zayakova and colleagues reported an increasing mortality rate trend in their study from 2007 to 2011,^34^ and some studies reported a constant trend in the mortality rate.^23^ Nonetheless, a general decreasing trend was reported in most studies which can be related to multiple factors such as the implementation of safety programs, improvement of workplace safety,^7^ upgrades on the training of prevention of burns and avoiding flammable objects, advances in treatment methods and techniques such as anti-microbial dressings for the burned areas of the body, improvement in intensive care, organizing instructional campaigns, and improvement of the medical equipment.^20^ Moreover, it should be considered that the preventive measures that decrease incidence would consequently lower the mortality rate.

 In our study, the total YLL were 25,260 in men and 25,785 in women, with the highest incidence pertaining to the 15-29 age group and the lowest incidence to the 80 + age group. Because life expectancy is taken into account for calculating YLL, it is reasonable that the lowest YLL are seen in the elders who have a lower life expectancy. Also, a decreasing trend in the YLL in both sexes was seen in our study, the decreasing trend of YLL was more pronounced in women than men (APC -9.2% vs. -5.8%). Similar to our results, a study recently published by Sadeghian and colleagues stated that during the 26-year study, a decreasing trend in the YLL was seen. Also, the APC of YLLs in this study was reported at -4.3% for women and -0.3% for men.^35^ The available evidence can indicate that women follow burn prevention instructions more than men. Of course, we should not ignore that men are at greater risk of burns than women because of their jobs. In addition, the improvement of treatment and care services for burn patients can play an important role in this reduction.

 The results from the joinpoint regression in our study showed that the annual change in mortality rate was -5.8% in men, -9.2% in women, and -7.8% in total. Another study reported a yearly change percentage of -5.4% in men and -4.2% in women.^7^ As mentioned before, there are numerous factors involved in this reduction. However, it should be considered that different studies used people from different age groups and varying stages of burns, making comparisons difficult. Furthermore, the role of cultural factors, economic factors, age distribution, and gender distribution of societies should not be neglected.^36^ Another critical factor is the availability of health care services which can affect mortality in different countries.^36^

 Some limitations of this study include the possibility of undercounting the cases of death due to burns, whereas some of the strengths of the study are the wide range of time and the appropriate sample size. This study is one of the few studies which analyzes the trend of the YLL due to burning.

 In conclusion, according to our findings, although the trend of YLL and mortality decreased, the highest number of deaths occurred in the 15-29 age group which consists of productive labor forces. In order to reduce injury, policymakers should develop necessary strategies for preventive measures, provide protective equipment in the workplace, and improve the accessibility of medical supplies and equipment for immediate outpatient treatment in high-risk workplaces. Training guidelines for educating women at risk should also be considered. Appropriate intervention measures should be taken in this regard. It is also suggested that more research should be conducted on the factors affecting burns to develop more accurate strategies.
